# Targeted Transcriptome Analysis of Beef Cattle Persistently Infected with Bovine Viral Diarrhea Virus

**DOI:** 10.3390/genes15121500

**Published:** 2024-11-22

**Authors:** Morgan Adkins, Sonia Moisa, Jon Beever, Andrea Lear

**Affiliations:** 1 College of Veterinary Medicine, University of Tennessee, Knoxville, TN 37919, USA; madkin19@utk.edu; 2 Animal Science, University of Tennessee, Knoxville, TN 37919, USA; smoisa@utk.edu (S.M.); jbeever@utk.edu (J.B.)

**Keywords:** bovine viral diarrhea virus, persistent infection, interferon, beef cattle, immune response

## Abstract

Background: Bovine viral diarrhea virus (BVDV) is an endemic virus of North American cattle populations with significant economic and animal health impacts. While BVDV infection has a myriad of clinical manifestations, a unique and problematic outcome is the establishment of a persistently infected (PI) animal following in utero viral infection. While it is well established that PI animals serve as a constant reservoir of BVDV, the mechanism for the maintained infection remains unknown despite multiple theories. Objective: The purpose of this study was to use transcriptome analysis to investigate the long-term immune status of adult PI cattle and offer insight into the potential mechanistic establishment of persistent BVDV infection. Methods: Peripheral blood mononuclear cells were collected from PI beef cattle (N = 6) and controls (N = 6) for targeted RNAseq analysis using 54 immune-related genes followed by pathway enrichment analysis. Results: Analysis revealed 29 differentially expressed genes (FDR < 0.05, fold change ≥ 2), representing 14 significant KEGG pathways between groups (FDR < 0.05). Transcriptome changes indicated chronic upregulation of interferon-gamma (*IFNG*) with an unexpected expression of related genes. Conclusions: These results provide novel insight into understanding the adult PI immune system and indicate maintained stimulation resulting from virus-mediated dysregulation.

## 1. Introduction

Bovine viral diarrhea virus (BVDV) is an endemic pathogen causing significant economic losses between USD 400 million and USD 1.4 billion [1,2,3,4] per annum in the United States. These losses occur through increased morbidity and mortality and decreased animal performance [5]. BVDV belongs to the family *Flaviviridae* and the genus *Pestivirus* [6]. Pestiviruses are enveloped, single-stranded, positive-sense RNA viruses and include other food animal pathogens such as classical swine fever virus and border disease virus [7]. While cattle are the primary host of concern, BVDV is capable of infecting other artiodactyls, including small ruminants, camelids, deer, and swine [8]. Within cattle populations, there are two BVDV genotypes: BVDV-1 and BVDV-2 [9]. Recent nomenclature changes by the International Committee on Taxonomy of Viruses have redesignated the family into pestiviruses A-K [10]. Despite the new classification system, *Pestivirus A* and *Pestivirus B* continue to be referred to as BVDV-1 and BVDV-2, respectively. Within the two genotypes, sub-genotypes exist with 22 BVDV-1 (1a–1v) and 4 BVDV-2 (2a–2d) strains identified [11]. Within North America, the predominant sub-genotypes are BVDV-1a, BVDV-1b, and BVDV-2a [12]. Strains of BVDV can be further subdivided according to their biotype, cytopathic or noncytopathic, depending on their ability to cause cytopathic effects in cultured cells [13,14].

Challenges associated with BVDV are not only attributed to the multitude of strains but also to the numerous clinical manifestations associated with infection [15]. In immunocompetent cattle, the most common outcome of the disease is a transient, self-limiting infection with absent to mild clinical signs of diarrhea, low-grade fever, and cough [7]. Less commonly, cattle can suffer acutely from severe disease with thrombocytopenia, high fever, and high fatality [7]. BVDV is highly detrimental to the cattle industry through its role in Bovine Respiratory Disease Complex (BRDC) [16]. While BVDV is considered one of the significant pathogens of BRDC, it alone does not cause overt pneumonic disease but rather increases host susceptibility to secondary viral and bacterial pneumonia by causing host immunosuppression [9,17].

Reproductive consequences are the second category of clinical manifestations of BVDV infections with deleterious effects on cattle health and production. When a pregnant cow is acutely infected, BVDV can cause a wide range of outcomes for the pregnancy depending on the gestational timing of exposure [18]. Infection within the first 45 days of gestation can result in infertility and early embryonic loss that may be perceived as failure to conceive or reduced herd pregnancy rates [19], but abortion can continue throughout pregnancy. Outcomes of transplacental transmission resulting in fetal infection are dependent on the gestational age of the fetus, the fetal organ system infected, and the biotype of the viral strain [20]. Fetal infection with a non-cytopathic strain from days 45 to 125 of gestation will result in a persistently infected (PI) calf that may be born apparently healthy but will be considered ‘immunotolerant’ to the infecting strain and therefore unable to clear the virus [6]. Congenital abnormalities may occur if the fetus is infected during organogenesis from days 100 to 150 of gestation and present as cerebellar hypoplasia, cataracts, microencephaly, hypotrichosis, as well as other manifestations [12,20]. Fetal infection in the later stages of gestation, generally considered beyond 150 days, can result in a clinically healthy calf that was transiently infected in utero with the ability to seroconvert and clear the viremia prior to birth [12].

While the prevalence of PI calves is low, estimated to be less than 1% in United States cattle populations [14], they provide a constant source of viral exposure and infection of immunologically naive animals to allow for the establishment of continuous BVDV infection within cattle populations [20]. Since PI animals are unable to develop a productive immune response to the infecting strain, they will shed large amounts of virus in all secretions, including milk, saliva, tears, nasal secretions, urine, blood, and trans-placentally to unborn calves [21]. Although PI animals may appear clinically normal and survive into adulthood, they are generally considered to be more susceptible to secondary infections attributed to ill-thrift and impaired immune function [22]. A catastrophic outcome for a PI animal is the development of mucosal disease, which results in severe erosions and ulceration of tissues, particularly in the gastrointestinal tract, whenever the animal becomes infected with a cytopathic strain that is antigenically similar to the previously tolerated, non-cytopathic strain [7].

One theory regarding the development of a PI animal is viral avoidance of the innate immune system, specifically through the lack of a productive interferon (IFN) response [7,23,24]. However, more recent work has demonstrated the ability of BVDV infection to elicit a host immune response during the creation of a persistent infection in a fetus [11]. Following a peak of maternal immune response and subsequent resolution of maternal viremia, PI fetuses display an acute and robust upregulation of interferon-gamma (*IFNG*) during fetal peak viremia along with upregulation of interferon-induced genes such as signal transducer and activator of transcription 1 (*STAT1*), transporter 1, ATP binding cassette 1 (*TAP1*), IFN-induced protein 16 (*IFI16*), chemokine ligand 10 (*CXC10*) and 16 (*CXC16*), and CXC receptor 6 (*CXCR6)* [24]. To further investigate the potential effects of maternal influence on the PI fetus immune response, peripheral blood mononuclear cells of dams were shown to have an upregulation of IFN activity at peak maternal viremia with a return to baseline prior to fetal IFN activation, which indicates that fetal interferon activity is not secondary to ongoing maternal immune activation [25]. Furthermore, immune activity has been demonstrated beyond the fetal period by demonstrating an upregulation of the interferon pathway in neonatal PI calves compared to uninfected cohorts [26]. Despite evidence of an active immune response, the fetal immune system is unable to clear the virus, which allows for the establishment of a persistent infection.

The mechanism of immune evasion, or lack of viral clearance, resulting in a persistently infected calf with BVDV continues to be unknown. Multiple theories speculate on the mechanism, including the inhibition of a type I interferon response that allows for ongoing viral persistence and replication or viral incorporation into the host T-cell repertoire, resulting in immunotolerance from the adaptive immune system [27]. Despite theories of PI establishment existing as the current narrative to explain this unique clinical outcome, the true mechanism of established infection has yet to be elucidated. Evaluation of adult PI animals provides the opportunity to explore impacts on the immune system that indicate a permanent alteration to function and activity. To further explore the mechanism of PI infection and the long-term impacts of maintained BVDV infection, this present study aims to use targeted RNAseq to investigate differentially expressed genes (DEGs) in persistently infected adult cattle compared to control animals. Furthermore, the goal is to further identify BVDV-host interactions by identifying signaling pathways that are upregulated or suppressed in the PI animals. The hypothesis is that there will be significant gene expression changes and altered signaling pathways of the persistently infected animals. This study characterized differential gene expression of adult cattle persistently infected with BVDV and demonstrated alterations of *INFG* activity and host antiviral pathways.

## 2. Materials and Methods

### 2.1. Ethics Statement

Animal housing, use, and sample collection were reviewed and approved by the Institutional Animal Care and Use Committee (North Auburn Beef Unit SOP 2020-3650) at Auburn University College of Veterinary Medicine, Auburn, AL, USA).

### 2.2. Animals and Sampling

Blood samples were collected for isolation of peripheral blood mononuclear cells (PBMC) from the coccygeal vein of six previously diagnosed PI cattle (mean age: 28.77 months, SEM: 5.189) based on antigen capture ELISA of skin notches and six control animals (mean age: 49.38 months, SEM: 10.44) based on convenience sampling (Supplementary Table S1). All but two animals reside in a university-owned herd in separate pastures with no fence line contact. One yearling calf was identified in Knoxville, TN, through routine herd screening, and an age-matched control was selected from the same location for sample collection. Ten milliliters of whole blood was collected in EDTA vacutainer tubes and stored on ice for six hours while transported to the lab for processing.

### 2.3. Mononuclear Lymphocyte Isolation from Whole Blood

Each whole blood sample was transferred into a sterile tube and diluted 1:1 with phosphate-buffered saline (PBS) without magnesium/calcium at room temperature conditions. Ten milliliters of LSM^®^ (Lymphocyte Separation Medium) (MP Biomedicals, LLC, Santa Ana, CA, USA) was added into a 50 mL conical tube, and the diluted blood solution was carefully layered overtop without mixing. The samples were centrifuged at 20 °C for 30 min at 700× *g*. Following centrifugation, the top layer of plasma was aspirated and discarded without disrupting the lymphocyte layer. The lymphocyte layer was collected into a clean tube with minimal aspiration of the red blood cell layer below. An equal volume of PBS was added to the collected lymphocyte layer, and samples were centrifuged at 20 °C for 10 min at 200 *×g* to wash lymphocytes and allow the elimination of LSM and additional platelets. One milliliter of 1x Red Blood Cell Lysis Buffer (10× Red Blood Cell Lysis Buffer, BioVision, Milpitas, CA, USA) was used to resuspend the lymphocyte pellets, and samples were incubated for 10 min at room temperature to lyse the red blood cells. Samples were then centrifuged at 20 °C for 10 min at 200× *g*, and supernatant was removed without disturbing the cell pellet. Cell pellets were resuspended in commercially available cell freezing media (Bambanker Standard, Bulldog-Bio Cryopreservation Reagents, Portsmouth, NH, USA) and then transferred to cryovials to be frozen at −80 °C per manufacturer’s recommendations.

### 2.4. RNA Isolation, Quantification, and Qualification

RNA was extracted from frozen PBMC pellets using TRIzol according to the manufacturer’s recommendations. RNA pellets were resuspended in 20 uL of 1:40 RNAse inhibitor/RNAse-free water mix and stored at −80 °C. A NanoDrop C Spectrophotometer was used for RNA quantification, measuring absorbance at 260 nm. Samples were assessed for quality based on AD 260/280 ratios of ≥1.9 and OD 260/230 ≥ 2.0. Finally, quality of RNA samples was confirmed using a Tapestation 4200 (Agilent, Inc., Santa Clara, CA, USA). RNA samples having a RIN > 7 were used for subsequent analysis.

### 2.5. cDNA Preparation

For the synthesis of cDNA, LunaScript^®^ RT SuperMix Kit was used following manufacturer’s recommendations (Cat#E3010L, New England BioLabs, Ipswich, MA, USA). Briefly, up to 1 µg total RNA per sample in combination with 4 µL of RT Supermix (5×) was used for a final volume of 20 µL. Reverse transcription-PCR was conducted in triplicate for cDNA synthesis. The incubation reaction consisted of a primer annealing step of 2 min at 25 °C, a cDNA synthesis step of 10 min at 55 °C, and a final heat incubation of 1 min at 95 °C. Expression values for each gene were normalized to the geometric mean of three housekeeping genes (*ACTB*, *YHAZ*, and *TBP*). Normalized reads for each sample triplicate were averaged and used for statistical analyses.

### 2.6. Primers Design for Target RNAseq

Individual primer pairs were designed using Batch Primer 3 (http://probes.pw.usda.gov/batchprimer3/ (Accessed on 28 March 2022)) at default settings for generic primers with total amplicon size set as an optimum of 60 to 80 bp. Primer sequences are listed in Supplementary Table S2. The primer sequences and cDNA were submitted to Floodlight Genomics (FG, Knoxville, TN, USA) for processing through the Educational and Research Outreach Program (EROP) using an optimized Hi-Plex targeted sequencing approach [28]. The Hi-Plex approach pools primers to PCR amplify targets and adds a barcode sequence during the amplification process. The resulting target library was then sequenced on an Illumina HiSeqX device at Admera Health LLC (South Plainfield, NJ, USA). The sample-specific FASTQ files were delivered via a secure data link for further processing.

### 2.7. Targeted RNA Sequencing Analysis

A targeted RNA sequencing approach was used to quantify expression levels in PBMC from the coccygeal vein of previously diagnosed PI calves compared to controls. Primers were selected to produce amplicons that span exon–intron boundaries using gene models extracted from Ensembl (www.ensembl.org (Accessed on 30 March 2022)) based on the cow genome assembly ARS-UCD1.2. The primer set information is provided in Supplementary Table S2. After validating the primers set, cDNAs were amplified in triplicates and sequenced using the same multiplex approach. Targeted RNA-seq was performed by an optimized Hi-Plex approach by Floodlight Genomics (Knoxville, TN, USA) as part of their Educational and Research Outreach Program (EROP) and sequencing on an Illumina MiSeq device running a 2 × 150 configuration [28]. The resulting sequences were mapped to the Bos Taurus genome assembly using CLC Genomics Workbench version 9.5.2 (Qiagen, Bethesda, MD, USA) with default settings. Multidimensional scaling (MDS) showed a separation in PI status compared to the control animals with the remove unwanted variation factor (RUV) set to 4.

Raw sequence reads were aligned to the cow reference genome (Bos taurus ARS-UCD1.2) using STAR [29] and counted using HtSeq [30].

A total number of 2,986,548 read pairs were generated for the 36 libraries, with sequencing yields ranging from 8015 to 221,822 per sample [28]. The read quality was checked by FastQC v11.5 [31]. Sequencing adapter sequences and low-quality bases were trimmed using Trimmomatic v0.3 [32]. The high-quality reads were mapped to the cattle reference genome (GenBank: GCA_002263795.2) using Salmon 1.10.2 to quantify the expression [33].

### 2.8. Differential Gene Expression Analysis

The DESeq2 package in R was used to normalize read counts and detect differentially expressed genes (DEGs) at a False Discovery Rate (FDR) threshold of 0.05. The log2 fold change (LogFC) was determined for each gene. The dataset analyzed during the current study is available in the NCBI Gene Expression Omnibus https://www.ncbi.nlm.nih.gov/geo/ under accession number GSE 278855 (Accessed on 31 December 2024).

### 2.9. Functional Annotation of Genes

Functional annotation of genes was carried out to gain insight into the underlying biology of the effect of BVDV in mononuclear lymphocytes of PI calves. Database for Annotation, Visualization, and Integrated Discovery (DAVID, version 6.8) [34] was used for functional annotation. DAVID assigned genes to pathways as per the Kyoto Encyclopedia of Genes and Genomes (KEGG) and determined enrichment of pathways using Fisher’s exact test [35]. In order to account for multiple tests, a Benjamini–Hochberg correction was applied [36]. A list of DEGs was generated using FDR < 0.05 as a cutoff value. KEGG pathways and gene ontology terms were deemed to be significant if they obtained a corrected *p*-value of <0.05.

## 3. Results

To investigate alterations in immune expression induced by BVDV PI status, cellular gene expression was analyzed using targeted RNAseq with 54 genes of interest based on expected immune activation or activity associated with BVDV infection (Supplementary Table S2). Analysis revealed 29 transcripts differentially expressed (*p* < 0.05, FDR < 0.05, fold change ≥ 2) (Table 1).

**Figure 1:**
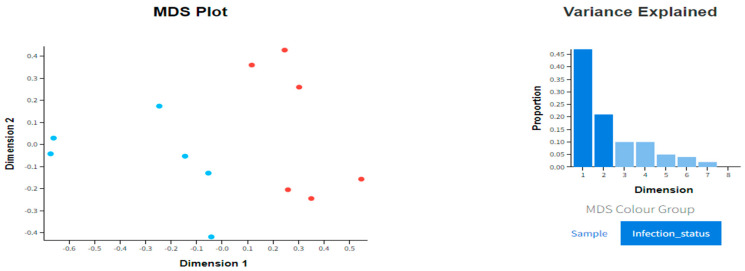
Multidimensional scaling plot and variance explained histogram. Blue dots represent persistently infected animals, and red dots represent control animals; MDS plot was used to demonstrate a visualization of similarities between animals of infection status based on DEGs with a variance histogram to demonstrate proportion of unwanted variation in dataset.

The top five upregulated genes in PI cattle were *IL10*, *AFT3*, *IFNG*, *CCL4*, and *CCL3*, while the five most downregulated genes included *OAS1Z*, *CXCR6*, *OAS1X*, *GBP5*, and *IFI35*. Significant differentially expressed genes revealed alterations related to an interferon response in persistently infected animals. Multidimensional scaling (MDS) showed a separation between PI cattle and control cattle based on targeted RNAse gene expression (Figure 1).

DAVID was used for the analysis of GO and enriched molecular pathway analysis and revealed 14 significant KEGG pathways between PI and control groups (FDR < 0.05) (Table 2). The top six most significant pathways sharing seven common DEGs included Hepatitis C, Influenza A, Chemokine signaling pathway, NOD-like receptor signaling pathway, Human cytomegalovirus infection, and Coronavirus disease—COVID-19 (FDR < 0.05). These genes had two enriched biological process terms and one cellular component term: inflammatory response, positive regulation of inflammatory response, and cytoplasm (FDR < 0.05).

## 4. Discussion

The present study evaluated changes in gene expression in peripheral blood mononuclear cells isolated from whole blood of adult cattle persistently infected with BVDV. Targeted RNA-seq was performed with a focus on immune function with the goal of identifying DEGs and altered activity of gene networks to provide further insight into the long-term consequences of persistent infection caused by fetal exposure to BVDV. When compared to uninfected cohorts, adult PI animals demonstrated ongoing activation of an *INFG* response and viral-mediated pathways, providing evidence of long-term immune activity in response to BVDV infection.

Results of this current study provide insight into IFN-γ activity in adult PI cattle that allude to a chronic immune response to viral presence despite an inability to achieve viral clearance. It has been theorized that interference of the interferon cascade may be a component in the establishment of persistent infection by allowing BVDV to evade the immune system [26]. The interferon cascade is divided into two main classes, type-I IFNs and type-II IFNs, which have broad biological functions, including antiviral, antiproliferative, and immunomodulatory effects through the JAK-STAT pathway, IFN-activated STATs, and CRK family of adaptor proteins [37,38]. When compared to cohorts on the same farm, adult PI animals in this current study demonstrated alterations of *IFNG* and related genes, which indicates ongoing immune activation likely to the presence of BVDV years after fetal exposure. Analysis revealed upregulation of *IFNG* with altered expression of multiple interferon-related genes, including downregulation of *OAS1Z*, *OAS1X*, *GBN5*, *IFI35*, *MX1,* and *IRF3* and upregulation of *IFI16* and *IL10*. Findings of downregulated genes responsible for antiviral activity in the face of an upregulated *IFNG* response were an unexpected outcome; however, similar results have been demonstrated in cell culture. An in vitro study of MDBK cells by Liu et al. [39] demonstrated the downregulation of antiviral-related genes, including *ISG15*, *MX1*, and *OAS1Y*. These findings support the role of IFN in the establishment of PI animals and may indicate dysregulation of the INFG pathway.

IFN-γ has multiple functions, including overlapping antiviral activity with type-I IFNs, enhancement of antigen presentation by stimulating major histocompatibility complex class I and II expression, antibody isotype switching of B cells, activation of macrophages, and mediating adhesive properties of endothelial cells [40]. Through stimulation of antigen-presenting cells (APCs), IFN-γ serves a role in the activation of the adaptive immune response of both CD4+ helper T cells and CD8+ cytotoxic T cells. CD4+ helper T cells then function to release cytokines for B cells and further T-cell activation, while CD8+ cytotoxic T cells induce apoptotic actions of infected host cells [4]. Through these actions, IFN-γ serves a critical role in communication between the innate and adaptive immune response [41]. Georges et al. [4] also investigated changes to the IFN response of PI fetuses with altered regulation of ISGs, primarily *STAT4*, and lack of subsequent lymphocyte activation. As a linking component between the innate and adaptive immune response, increased IFN activity would be expected to result in an increase in the adaptive immune response and potentially successful viral clearance; however, this is not demonstrated in PI animals

Upregulation of *IFNG* in this population of animals demonstrates ongoing stimulation of the innate immune response, presumably in response to the infecting strain of non-cytopathic BVDV but with inappropriate mediation from an upregulation of *IL10*. *IL10* has multiple functions as a cytokine produced by various cell types, including B cells, macrophages, and CD4+ T cells [42]. Generally, IL-10 is considered immunosuppressive through the downregulation of MHC class II activities and, therefore, reduces the activation of T cells and inhibition of pro-inflammatory cytokine production by blocking the JAK-STAT pathway [43]. Reduced antigen presentation and impaired activation of T cells provide evidence for a lack of adaptive immune signaling activity that would allow for attempted viral clearance. In chronic infection models evaluating *Fasciola hepatica* in cattle, constant upregulation of *IL10* is associated with inhibition of *IFNG* [44], but in these PI animals, both genes were significantly upregulated, which should allow for IFN-γ priming of antigen presentation and activation of the adaptive immune response. The chronic upregulation of both *IL10* and *IFNG* together indicates recognition of the immune system of infection status but demonstrates an inappropriate response of immune activation. These findings together suggest that BVDV does not only stimulate immune activity through upregulation of IFN activity but may inhibit host antiviral activity and impede immune function through dysregulation of the IFN-related genes and appropriate immune activation.

Comparisons of DEGs of this current study to transcriptome analysis of cattle acutely infected with Peste des petits ruminants virus (PPRV) demonstrate the unexpected expression of the interferon-related genes in PI infection [45]. In cattle infected with PRRV, 22 genes were upregulated, demonstrating host-mediated antiviral activity, including *IFNG*, *OAS1X*, *OAS1Y*, *MX1*, and *IFI6,* along with other interferon-related genes. The upregulation of all these genes demonstrates a reactive and coordinated response of the type-II interferon pathway in response to viral infection [45]. The chronic upregulation of *IFNG* in the PI cattle, paired with inappropriate upregulation of *IL10* and downregulation of most interferon-related DEGs, highlights potential dysfunction or altered activity of the PI immune system. These findings may indicate an unidentified negative feedback mechanism, immune exhaustion secondary to constant viral stimulation [46], or inappropriate immune signaling. These potential mechanisms may explain a maintained innate stimulation with a lack of correspondence and appropriate modulation of an adaptive immune response necessary for viral clearance.

Two of the most significantly downregulated genes in this current study were *OAS1Z* and *OAS1X.* These genes encode proteins that are induced by IFN activity and have significant antiviral properties, including degradation of viral RNA and activation of cytoplasmic pattern-recognition receptors, including RIG-1 and MDA-5 [47]. The primary mechanism of the OAS pathway is the stimulation of OAS enzymatic proteins following host cell viral RNA exposure and IFN activation [48]. OAS then activates RNase L, which cleaves both viral and cellular RNA, which is one of the pathway’s antiviral mechanisms. Once viral RNA is cleaved, MDA-5 and RIG-1 will promote the activation of interferon-stimulated genes *IRF3* and *IRF7* [49]. In the PI animals, however, there was significant downregulation of *OAS1Z*, *OAS1X*, and *IRF3* despite ongoing viral exposure and upregulation of *IFNG*. In a normally functioning immune response, increased OAS activity should pair with an increase in interferon-stimulating genes and interferon activation. The findings in this study provide further evidence of chronic inappropriate innate immune function due to viral interference with the IFN pathway. Furthermore, inappropriate interferon activity may indicate an undefined malfunction that prevents coordination and communication between the innate and adaptive immune response, providing a mechanism for lifelong PI status. Results from this study mirror findings from Nilson et al. [50], who demonstrated a chronic type I IFN response in PI animals with fewer alterations of the adaptive immune system, leading to speculation that PI status leads to chronic IFN dysregulation and limited activation of the adaptive immune response.

The significant changes in DEGs of the PI cattle provide evidence that infection status has long-term effects on immune-related gene expression. Enrichment analysis provides a further understanding of signal transduction pathways mediated by persistent BVDV infection and provides additional information on the host immune response to viral infection. The top five most significantly enriched KEGG pathways were Hepatitis C, Influenza A, Chemokine signaling pathway, NOD-like receptor signaling pathway, and Human cytomegalovirus infection, which are all defined as viral response or reactive immune signaling pathways. Genes involved in these pathways include *OAS1XZ*, *RAF1*, *IFN-γ*, *MX1*, *STAT3*, *IRF3*, *CXCL8*, *OAS1X*, *CCL3*, *GNB1*, *CCL4*, *CXCR6*, *GBP5*, *IFI16*, and *CASP4*. Many of the overrepresented pathways in the PI animals involved host antiviral response and innate immune function. While many of the pathways are named according to human viral diseases of importance, such as Hepatitis C, COVID-19, and Measles, the enrichment of the DEGs indicates the activities of these pathways being involved in a myriad of potential viral infections. DEGs of pathway enrichment in this study overlap with findings of other transcriptome analyses exploring other pathogens of BRDC. An evaluation of BRDC pathogens of bronchial lymph nodes identified pathways related to innate immune response [51], with subsequent studies identifying pathways overlapping with this transcriptome analysis, including Influenza A and pathways involving chemokine activity [52]. Most similarly, whole blood transcriptome analysis in dairy calves infected with bovine herpesvirus 1 and bovine respiratory syncytial virus identified enriched KEGG pathways including Influenza A, cytokine–cytokine receptor interaction, and NOD-like receptor signaling [53]. Finding similarities with transcriptome analysis between the PI animals and those with acute BRDC viral infections offers additional compelling insight into the PI immune system and verifies recognition of the immune system to the maintained viral infection. The enrichment of pathways involving host response to viral infection demonstrates the ongoing stimulation and, therefore, recognition of the PI immune system of the permanently established BVDV pathogen. Evidence suggests that the PI host endures chronic response to the viral infection but lacks the ability to appropriately succeed at viral clearance. Demonstrating evidence of lifelong immune activation of PI cattle changes the narrative of BVDV persistent infection and offers a direction for further investigating the mechanism of infection status and host interaction.

While the findings of this work demonstrate a novel evaluation of adult PI immune status through transcriptome analysis, it also supports previous findings of PI immune function and fetal immune characteristics. PI animals are generally considered to have poor immunological status, which further defines the narrative of altered immune function secondary to viral presence. Deleterious effects on PI immune function have been demonstrated through reduced neutrophil function, decreased lymphocyte blastogenesis, and reduced antibody titers in response to pathogen exposure [14]. PI dairy calves have also been demonstrated to maintain an elevation in haptoglobin levels, an acute phase protein, indicating an ongoing pro-inflammatory response [54]. Microarray analysis of yearling PI steers has identified a robust and seemingly chronic antiviral response through the upregulation of various interferon-stimulating genes (ISGs), including *ISG15* and *OAS-1* [26]. Long-term derangements of the PI immune system have been demonstrated in this work and others, with previous transcriptome analysis demonstrating changes in gene expression of multiple interferon-related genes starting in utero [4,24,25,26,27]. The findings of this transcriptome analysis demonstrate ongoing changes within the PI immune system, with evidence indicating alterations in utero with derangements over the lifetime.

A limitation of this study was the lack of uniformity in the cattle population. The variation of breed, age, and sex of the cattle reflects a convenience population of PI animals that have been previously identified and maintained in a university setting. The cattle were collected from various sources through incidental diagnosis when screening for PI infection in herds, as opposed to establishing PI infection in bovine fetuses through maternal virus inoculation of a non-cytopathic strain of BVDV or purposeful exposure of pregnant dams to a PI animal. Ideally, these animals would have been a more uniform group of animals to reduce variation in genetic makeup, immune function, epigenetic alterations to gene expression, animal location, and virulence characteristics of the BVDV strain. The convenience sampling of this study population was constrained by adult PI animal availability and may introduce sample selection bias into this study. However, the heterogeneous nature of the animals may provide strength to the analysis by demonstrating DEGs and enriched pathways despite the variation within the sample population. Additionally, the non-cytopathic variants of BVDV of each animal’s infection were not genotyped, and given that cattle were infected via wild-type exposure, it can be assumed that strains of BVDV were genetically heterogeneous. Genomic variation of BVDV may demonstrate variance in pathogenicity and induce differing degrees of immune activation. While a single primary strain of BVDV may induce a more uniform response, the variation in the strains demonstrated significant alterations to the transcriptome of the PI cattle of this study population and provides evidence of maintained immune activation. Genetic variation may also provide an explanation for the variation in pathway activation between this study of adult PI animals with primary immune activity involved a type-II interferon response compared to Nilson et al. [50], where dysregulation was attributed to a type-I interferon response. A more robust analysis would include global transcriptome analysis over targeted analysis. Genes selected for evaluation in this data set were selected based on previous findings, primarily with PI fetal tissues. Genes selected were evaluated with the expectation of identifying DEGs; however, not all selected genes demonstrated altered expression, and pathway analysis provided a dynamic evaluation of immune function to avoid the introduction of confirmation bias into this study.

## 5. Conclusions

In this study, we characterized gene expression through targeted RNA-seq of adult cattle persistently infected with BVDV to investigate the long-term consequences of the immune system secondary to this unique fetal infection. DEGs and enrichment pathways demonstrate a response of the PI immune system to the ongoing presence of the virus. The altered expression of IFN-related genes provides evidence that dysregulation of a type-II interferon response may be a component for the establishment of permanent PI status through a lack of stimulation of the adaptive immune system. With findings of these data demonstrating similar pathway enrichment compared to acutely viremic animals, this work provides evidence of PIs maintaining an ongoing antiviral response and activity, indicating immune recognition of the virus. This current study helps to further define the PI immune function and offers insight into virus-mediated effects, although the mechanism of maintained infection remains undefined. Future directions for this work would include global transcriptome analysis of adult PI cattle to evaluate additional DEGs and enrichment pathways to help further define chronic alterations to the PI immune system. To further explore the mechanism of PI establishment, epigenetic changes in immune-related genes could be investigated to offer an explanation for alterations in gene expression and immune function.

## Figures and Tables

**Table 1:** Summary of differentially expressed genes from targeted RNAseq by fold change of persistently infected animals compared to controls.

Gene ID	Entrez Gene ID	Gene Name	* Fold Change	*p* Value	FDR
*IL10*	281246	interleukin 10	2.13	1.8 × 10^−5^	0.001
*ATF3*	515266	activating transcription factor 3	1.86	2.6 × 10^−4^	0.002
*IFNG*	281237	interferon-gamma	1.21	4.8 × 10^−3^	0.014
*CCL4*	414347	C-C motif chemokine ligand 4	1.01	2.6 × 10^−2^	0.050
*CCL3*	282170	chemokine (C-C motif) ligand 3	0.96	7.2 × 10^−4^	0.004
*XAF1*	509740	XIAP associated factor 1	0.91	5.5 × 10^−5^	0.001
*XRCC5*	531945	X-ray repair cross-complementing 5	0.75	1.1 × 10^−2^	0.024
*HSF1*	506235	heat shock transcription factor 1	0.50	1.0 × 10^−3^	0.005
*COX11*	510509	cytochrome c oxidase copper chaperone COX11	0.46	2.9 × 10^−3^	0.009
*GNB1*	281201	G protein subunit beta 1	0.33	1.1 × 10^−4^	0.001
*HSPD1*	511913	heat shock protein family D (Hsp60) member 1	0.33	1.5 × 10^−2^	0.032
*EIF1*	509764	eukaryotic translation initiation factor 1	0.31	6.6 × 10^−4^	0.004
*CCNB1*	327679	cyclin B1	0.30	8.8 × 10^−3^	0.022
*CXCL8*	280828	C-X-C motif chemokine ligand 8	0.15	1.8 × 10^−3^	0.007
*CTDSP2*	506115	CTD small phosphatase 2	0.13	4.2 × 10^−3^	0.013
*IFI16*	506759	interferon-gamma-inducible protein 16	0.10	2.5 × 10^−3^	0.009
*IRF3*	516979	interferon regulatory factor 3	−0.06	1.2 × 10^−2^	0.026
*RPS17*	788861	ribosomal protein S17	−0.07	1.2 × 10^−3^	0.005
*PTGES3*	493638	prostaglandin E synthase 3	−0.15	7.0 × 10^−3^	0.020
*UBC*	444874	ubiquitin C	−0.16	5.7 × 10^−4^	0.004
*CASP4*	338039	caspase 4, apoptosis-related cysteine peptidase	−0.18	8.5 × 10^−5^	0.001
*STAT3*	508541	signal transducer and activator of transcription 3	−0.26	8.4 × 10^−3^	0.022
*MX1*	280872	MX dynamin like GTPase 1	−0.26	2.3 × 10^−2^	0.046
*RAF1*	521196	Raf-1 proto-onco, serine/threonine kinase	−0.34	9.6 × 10^−4^	0.005
*IFI35*	510697	interferon-induced protein 35	−0.35	1.1 × 10^−2^	0.024
*GBP5*	516949	guanylate binding protein 5	−0.37	2.7 × 10^−3^	0.009
*OAS1X*	347699	2′,5′-oligoadenylate synthetase 1, 40/46 kDa	−0.62	2.5 × 10^−4^	0.002
*CXCR6*	506807	C-X-C motif chemokine receptor 6	−0.62	9.7 × 10^−3^	0.024
*OAS1Z*	519922	2′,5′-oligoadenylate synthetase 1, 40/46 kDa	−1.09	1.6 × 10^−4^	0.002

* Fold change indicating gene expression between persistently infected cattle and control cattle. Green color represents activation or up-regulation, and red color represents inhibition or down-regulation, with intensity representing level of expression.

**Table 2:** Significant KEGG pathways and gene ontology terms identified using DAVID.

Category	Term	Count	%	*p* Value	FDR
KEGG_PATHWAY	bta05160:Hepatitis C	7	24	3.4 × 10^−6^	2.3 × 10^−4^
KEGG_PATHWAY	bta05164:Influenza A	7	24	5.6 × 10^−6^	2.3 × 10^−4^
KEGG_PATHWAY	bta04062:Chemokine signaling pathway	7	24	6.2 × 10^−6^	2.3 × 10^−4^
KEGG_PATHWAY	bta04621:NOD-like receptor signaling pathway	7	24	6.7 × 10^−6^	2.3 × 10^−4^
KEGG_PATHWAY	bta05163:Human cytomegalovirus infection	7	24	2.9 × 10^−5^	7.9 × 10^−4^
KEGG_PATHWAY	bta05171:Coronavirus disease—COVID-19	7	24	5.6 × 10^−5^	1.3 × 10^−3^
KEGG_PATHWAY	bta05167:Kaposi sarcoma-associated herpesvirus infection	6	21	1.9 × 10^−4^	3.7 × 10^−3^
KEGG_PATHWAY	bta05162:Measles	5	17	6.4 × 10^−4^	1.1 × 10^−2^
KEGG_PATHWAY	bta04060:Cytokine-cytokine receptor interaction	6	21	1.3 × 10^−3^	1.9 × 10^−2^
KEGG_PATHWAY	bta04061:Viral protein interaction with cytokine and cytokine receptor	4	14	1.7 × 10^−3^	2.3 × 10^−2^
KEGG_PATHWAY	bta05417:Lipid and atherosclerosis	5	17	2.8 × 10^−3^	3.4 × 10^−2^
KEGG_PATHWAY	bta04620:Toll-like receptor signaling pathway	4	14	3.0 × 10^−3^	3.4 × 10^−2^
KEGG_PATHWAY	bta05142:Chagas disease	4	14	3.5 × 10^−3^	3.6 × 10^−2^
KEGG_PATHWAY	bta04068:FoxO signaling pathway	4	14	4.3 × 10^−3^	4.1 × 10^−2^
GOTERM_CC_DIRECT	GO:0005737~cytoplasm	14	48	2.5 × 10^−4^	1.5 × 10^−2^
GOTERM_BP_DIRECT	GO:0006954~inflammatory response	6	21	3.5 × 10^−5^	1.1 × 10^−2^
GOTERM_BP_DIRECT	GO:0050729~positive regulation of inflammatory response	4	14	1.2 × 10^−4^	1.8 × 10^−2^

See Supplementary Table S3 for more information.

## Data Availability

The dataset analyzed during the current study is available in the NCBI Gene Expression Omnibus https://www.ncbi.nlm.nih.gov/geo/ under accession number GSE 278855. (Accessed on 31 December 2024).

## Supplementary Materials

The following supporting information can be downloaded at https://www.mdpi.com/article/10.3390/genes15121500/s1. Supplementary Table S1: Animal information, infection status, and farm location; Supplementary Table S2: Gene sequence IDs and primer sequences; Supplementary Table S3: KEGG and gene ontology pathway information and results.
